# Accelerated DNA methylation age in adolescent girls: associations with elevated diurnal cortisol and reduced hippocampal volume

**DOI:** 10.1038/tp.2017.188

**Published:** 2017-08-29

**Authors:** E G Davis, K L Humphreys, L M McEwen, M D Sacchet, M C Camacho, J L MacIsaac, D T S Lin, M S Kobor, I H Gotlib

**Affiliations:** 1 Department of Psychiatry and Behavioral Sciences, Stanford University, Stanford, CA, USA; 2 Department of Psychology, Stanford University, Stanford, CA, USA; 3 Department of Medical Genetics, BC Children’s Hospital Research Institute, University of British Columbia, Vancouver, BC, Canada

## Abstract

Numerous studies have linked exposure to stress to adverse health outcomes through the effects of cortisol, a product of the stress response system, on cellular aging processes. Accelerated DNA methylation age is a promising epigenetic marker associated with stress and disease risk that may constitute a link from stress response to changes in neural structures. Specifically, elevated glucocorticoid signaling likely contributes to accelerating DNA methylation age, which may signify a maladaptive stress-related cascade that leads to hippocampal atrophy. We examined the relations among diurnal cortisol levels, DNA methylation age and hippocampal volume in a longitudinal study of 46 adolescent girls. We computed area under the curve from two daily cortisol collection periods, and calculated DNA methylation age using previously established methods based on a set of CpG sites associated with chronological age. We computed a residual score by partialling out chronological age; higher discrepancies reflect relatively accelerated DNA methylation age. We assessed hippocampal volume via T1-weighted images and automated volumetric segmentation. We found that greater diurnal cortisol production was associated with accelerated DNA methylation age, which in turn was associated with reduced left hippocampal volume. Finally, accelerated DNA methylation age significantly mediated the association between diurnal cortisol and left hippocampal volume. Thus, accelerated DNA methylation age may be an epigenetic marker linking hypothalamic–pituitary–adrenal axis dysregulation with neural structure. If these findings are replicated, the current study provides a method for advancing our understanding of mechanisms by which glucocorticoid signaling is associated with cellular aging and brain development.

## Introduction

There is growing interest in understanding the pathways by which stressful experiences affect the aging process, including recent studies of molecular markers of aging, such as epigenetic modifications. One such epigenetic marker, methylation of 5′-cytosine-phosphate-guanine-3′ sites across the genome, has the potential to influence gene expression. Variation in DNA methylation (DNAm) has been found to be associated with aging and with mental and physical health difficulties.^1, 2, 3^ Researchers have developed multivariate predictors based on the methylation status of certain CpG sites in order to calculate estimates of chronological age.^4, 5^ One specific predictor is capable of accurately estimating DNAm age across several tissues including saliva.^4^ This DNAm age metric has been shown to be one of the most accurate biological measures of chronological age reported to date.^4^ Importantly, investigators have now documented discrepancies between DNAm age and chronological age, referred to as epigenetic age acceleration, as predictive of all-cause mortality^6, 7, 8, 9^ and associated with both stress exposure and the development of psychiatric disorders such as post-traumatic stress disorder (PTSD).^10, 11, 12^ These findings indicate that accelerated epigenetic age may be a biomarker of changes in stress-related cellular functioning.^13^

Leveraging data from a longitudinal study of familial risk for major depressive disorder (MDD), we quantified DNAm from saliva to estimate DNAm age^4^ in a sample of 46 healthy adolescent girls with or without a maternal history of MDD. Participants also provided diurnal cortisol samples and completed magnetic resonance imaging (MRI) scans to assess neural structure. Given that stress has been found to be associated with increased levels of diurnal cortisol in adolescence,^14^ accelerated methylation aging^10, 13^ and reduced hippocampal volume,^15^ we examined the associations among these three variables. We posited that diurnal cortisol levels would be associated with accelerated DNAm age which, as a biomarker of stress due to increased cortisol exposure, would in turn predict reduced hippocampal volume and mediate the association between diurnal cortisol level and hippocampal volume. In secondary analyses to investigate the specificity of this effect to volume of the hippocampus, we repeated the analyses using amygdala volume, another hypothalamic–pituitary–adrenal axis subcortical region responsive to stress but without consistent evidence of glucocorticoid-induced atrophy.^16, 17^ Given the recruitment procedures and longitudinal study design, we also examined whether these associations were moderated by either maternal history of depression or by the development of MDD during the course of the study.

## Materials and methods

Participants were selected to be at either high or low familial risk for depression based on maternal history of MDD. Specifically, mothers either had at least two episodes of MDD during the child’s life, or had no current or past MDD diagnosis. Adolescent girls were included only if they had no current or past MDD both at initial assessment and at the time the saliva sample was obtained for DNAm analysis. MDD was assessed using the Kiddie Schedule for Affective Disorders^18^ and participants were rescreened annually for the development of MDD (for more details see ref. 19). The 46 girls in this sample included 24 with high familial risk (mean age 12.87, s.d.=1.31) and 22 with low familial risk (mean age 12.14, s.d.=1.36) who were matched on age. The majority reported their race/ethnicity as Caucasian (61%), followed by multiracial (33%), African American (2%), Latina (2%) and Asian (2%). Participants provided four daily saliva samples over 2 days for assaying diurnal cortisol. Either at the same time or at a later date, these girls provided a saliva sample for DNAm analysis and completed an MRI scan to obtain T1-weighted structural images of their brain. Only girls with complete and usable data for all three measures were included in this study, and the sample size was not selected to ensure power for a pre-specified effect size. DNAm samples were obtained an average of 0.30 years after the diurnal cortisol sampling (s.d.=0.61; range 0–2.80). Participants completed MRI scans in an average of 4.08 years after the saliva samples for DNAm analysis were obtained (s.d.=2.80; range 0–9.11; Supplementary Table 1 for assessment ages). By the time of the MRI scan, 12 of the 46 girls (26%) had developed an episode of MDD; no girl was scanned during a depressive episode. All procedures were approved by the Stanford Institutional Review Board, and assent and informed consent were obtained from the participants and the participants’ parents, respectively.

### Diurnal cortisol methods

Over the course of 2 days, participants provided saliva samples using Salivette kits (Sarstedt, Germany) upon waking, 30 min after waking, midafternoon (~1500 hours) and 30 min before bedtime. Samples were stored frozen at −20 °C until assayed by luminescence immunoassay reagents according to commercial instructions (Immuno-Biological Laboratories, Hamburg, Germany). Total daily cortisol production was calculated as the area under the curve with respect to ground (AUCg)^20^ using exact time of sample collection. Outlying values (+2 s.d.) were corrected using winsorizing in the larger sample from the parent study (*N*=135).^19^

### DNA sample processing, DNAm microarray and DNAm age calculation

Saliva samples were collected using Oragene kits (DNA Genotek, Ottawa, ON, Canada), and genomic DNA was extracted using the DNeasy Kit (Qiagen, Hilden, Germany). The EZ DNA Methylation Kit (Zymo Research, Irvine, CA, USA) was used to bisulfite convert 750 ng of genomic DNA allowing for sequence-based differentiation of unmethylated and methylated cytosine nucleotides. Following the manufacturer’s instructions, an input of ~160 ng of bisulfite-converted DNA was used for whole-genome amplification and enzyme fragmentation, then hybridized to a MethylationEPIC BeadChip (Illumina, San Diego, CA, USA), a genome-wide platform that assesses over 850 000 CpG sites. Processed BeadChips were scanned on an Illumina HiScan and intensity values were imported into GenomeStudio (Illumina) for data quality assessment, color correction and background subtraction. A data matrix was then exported as beta values representing percent DNAm ranging from 0 to 1 (0=unmethylated and 1=methylated). This output was submitted to the online DNA Methylation Age Calculator (https://dnamage.genetics.ucla.edu)^4^ according to instructions, in order to obtain normalized DNAm age measures for each sample. Although the saliva samples consist of heterogeneous cell types, the DNA Methylation Age Calculator of DNAm age estimation has been shown to be robust to this source of variation.^4^ As can be seen in Supplemental Figure 1, we obtained the expected positive association between chronological age and DNAm age (*r*(46)=0.34,*P*=0.02). The size of the correlation is related to the restricted chronological age range of the sample (that is, a truncated range of chronological age reduces the ability to obtain large correlations with estimated DNAm age), and in fact, 83% of the DNAm samples deviated by <3.6 years from chronological age, a threshold determined by test samples validating the accuracy of the DNA Methylation Age Calculator.^4^ In addition, because DNAm age has been found to vary by ethnic group,^21^ we tested for differences as a function of reported race/ethnicity. Caucasian participants did not differ significantly from other/multiracial participants in DNAm age (*t*(44)=0.20, *P*=0.85). Taken together, these results indicate that the DNAm age estimation metric functions well in this sample and that our results are not confounded by race/ethnicity.

### MRI data acquisition

MRI scans were acquired at the Lucas Center (*N*=21) and the Center for Cognitive and Neurobiological Imaging (CNI; *N*=25) at Stanford University. The Lucas Center scans were obtained using a 1.5 T Signa Excite MR system (GE Healthcare Systems, Milwaukee, WI, USA) equipped with a 8-channel head coil, and structural images were obtained using a T1-weighted spoiled gradient-recalled echo sequence with the following parameters: sagittal slice orientation; number of slices=116; repetition time (TR)=8.92 ms; echo time (TE)=3.00 ms; inversion time (TI)=300 ms; flip angle=15° in-plane resolution=0.86 × 0.86 mm; slice thickness=1.5 mm; scan duration=645 s; and matrix size=256 × 192. The CNI T1-weighted scans were obtained using a 3 T Discovery MR750 MR system (GE Medical Systems, Milwaukee, WI, USA) equipped with a 32-channel head coil using an spoiled gradient-recalled pulse sequence: sagittal slice orientation; number of slices=186; TR=6.24 ms; TE=2.34; TI=450 ms; flip angle=12° voxel size=0.9 × 0.9 × 0.9 mm; scan duration=315 s; and matrix size=256 × 256. Given that we used two scanners, analyses included this variable as a dummy-coded covariate (0 vs 1). In Supplementary Table 2 we present a comparison of participant demographic information and key variables of interest across the two scanners.

### Brain volume segmentation

Automated segmentation of subcortical volumes (bilateral hippocampus and amygdala) and estimation of total intracranial volume from the T1-weighted images were obtained via the FreeSurfer software suite (v5.3; available at: http://surfer.nmr.mgh.harvard.edu/^22^). This approach has been shown to be robust to anatomic variability, and to have comparable accuracy to manual labeling techniques^22, 23^ and acceptable scan-rescan reliability.^24^ Using the FreeView image viewer, all subcortical volumes were visually inspected for major segmentation errors; 4/92 hippocampal segmentations and 0/92 amygdala segmentations were excluded.

### Data analysis

We created a set of unstandardized residual variables to interpret our three constructs of interest – cortisol AUCg, DNAm age and brain volume – by partialling out chronological age at sample collection/scan for each of these variables in addition to total intracranial volume when computing the residual subcortical volume scores (bilateral hippocampus and amygdala). These scores did not differ significantly by scanner; a repeated measures analysis of variance including structure (hippocampus and amygdala) and hemisphere (right and left) did not yield a main effect of scanner on estimated volumes (*F*(1,40)=2.52, *P*=0.12). We used ordinary least squares regression to examine associations among variables of interest. We conducted a single-step mediation analysis using 1000 bootstrap resamples and, given the preliminary nature of this study, calculated 90% confidence intervals to test for indirect effects.^25^

## Results

### Diurnal cortisol and DNAm age

**Figure 1:**
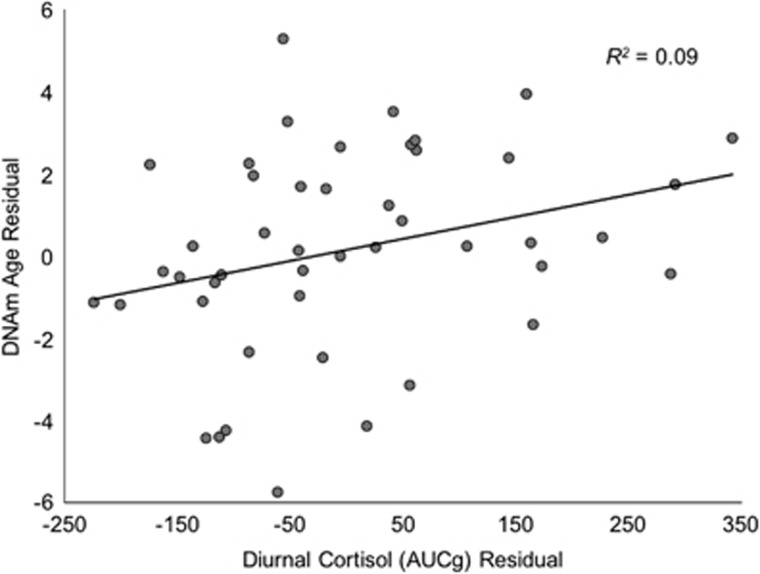
Diurnal cortisol production (area under the curve with respect to ground; AUCg) is associated with accelerated DNA methylation (DNAm) age.

Residual cortisol AUCg was significantly positively associated with DNAm age residual (*B*=0.01 [0.003], *β*=0.29, *t*(44)=2.02, *P*=0.049, Δ*R*^2^=0.09; Figure 1).

### DNAm age and bilateral hippocampal volume

**Figure 2:**
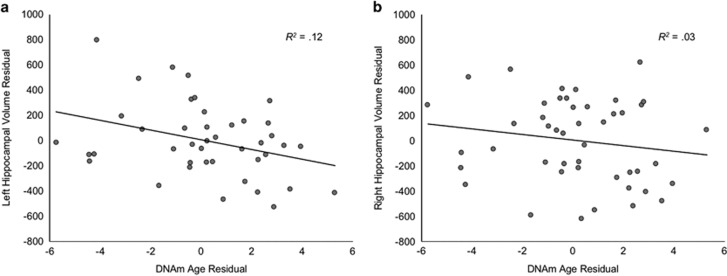
Accelerated DNA methylation (DNAm) age is associated with reduced (**a**) left but not (**b**) right hippocampal volume. Note: scanner included as a covariate.

DNAm age residual was significantly negatively associated with left hippocampal volume (*B*=−40.32 [17.42], *β*=−0.35, *t*(39)=−2.31, *P*=0.026, Δ*R*^2^=0.12; Figure 2a), controlling for scanner. The association was in the same direction, but not statistically significant, for right hippocampal volume (*B*=−23.25 [20.62], *β*=−0.16, *t*(43)=−1.13, *P*=0.27, Δ*R*^2^=0.03; Figure 2b).

### Diurnal cortisol and hippocampal volume

There was a trend-level negative relation between residual cortisol AUCg and left residual hippocampal volume (*B*=−0.66 [0.33], *β*=−0.30, *t*(39)=−1.99, *P*=0.054 and Δ*R*^2^=0.09; Supplemental Figure 2), and no association with right residual hippocampal volume (*B*=−0.54 [0.37], *β*=−0.21, *t*(43)=−1.48, *P*=0.15 and Δ*R*^2^=0.04), after controlling for scanner.

### Mediation: diurnal cortisol, DNAm age and left hippocampal volume

We conducted a mediation model with the subsample with available data for all three variables (*N*=42). Importantly, there was a significant indirect effect of residual cortisol AUCg levels on left residual hippocampal volume via accelerated DNAm age (coeff.=−0.18 [0.12], 90% confidence interval [−0.45, −0.03]). Further, this effect remained statistically significant (coeff.=−0.16 [0.12], 90% confidence interval [−0.41, −0.02]) when we conducted this mediation model with the subsample with available data in which the requirement for temporal ordering of the variables was met (that is, the sample for cortisol collection preceded the sample for DNAm analysis, and the MRI scan followed collection of the DNAm sample; *N*=39).

### DNAm age and bilateral amygdala volume

To evaluate the specificity of the above effect for the hippocampus, as a secondary analysis, we examined the association between DNAm age residual and bilateral amygdala volume. There were no significant associations between DNAm age residual and left (*P*=0.92) or right (*P*=0.68) amygdala volume.

### Associations with familial risk for depression and with the development of MDD

The participants with and without a maternal history of depression in this study did not differ on any of the key variables examined in these analyses (Supplementary Table 3); moreover, risk status did not moderate any of the observed bivariate associations or the full mediation (*P*s>0.05). In addition, the subset of girls who developed an episode of MDD after we obtained the saliva samples but before we conducted the MRI scan did not differ from participants who did not develop MDD in left or right hippocampal volume residuals (*t*(40)=−0.43, *P*=0.67 and *t*(44)=−0.28, *P*=0.78, respectively); moreover, MDD history did not moderate either the associations with hippocampal volume or the full mediation (*P*s>0.05). We should note here, however, that we are likely underpowered to detect significant moderations given the smaller sizes of these two subgroups.

## Discussion

Accelerated DNAm aging is associated with stress-related processes, and in particular with glucocorticoid-mediated alterations in gene expression.^13^ Deviations from age-expected methylation levels may be a link between stressful experiences and negative health outcomes.^10, 26^ In this study we documented in a sample of healthy adolescent girls who vary in their risk for MDD that elevated diurnal cortisol production is associated with accelerated DNAm age, which in turn is associated with reduced left hippocampal volume. Moreover, we demonstrated that accelerated DNAm age mediates the association between elevated cortisol and reduced left hippocampal volume, indicating that accelerated DNAm age may be an epigenetic marker linking hypothalamic–pituitary–adrenal axis dysregulation with neural structure.

DNAm across the genome^27^ and methylation levels within CpG sites in specific genes (for example, *SLC6A4, FKBP5, DNMT1*and*BDNF*)^28, 29, 30^ have been implicated in stress-related changes in hippocampal volume and localized gene expression in the hippocampus. The Horvath estimate of DNAm age^4^ includes a high proportion of epigenetic markers in glucocorticoid response elements;^13^ in the present study we found relatively increased estimates of DNAm age for individuals with higher levels of diurnal cortisol. In addition, we identified both direct and indirect paths linking cortisol and DNAm age, two biomarkers of stress, specifically with reduced hippocampal volume. Cortisol dysregulation has been posited to be a mechanism by which experiences of stress negatively affect hippocampal structure in the developing brain;^31^ the present findings are consistent with results of previous research linking cortisol production with hippocampal atrophy^15, 32^ and with volumetric reductions in specific subfields of the hippocampus.^33^ Due to the resolution of the MRI scans, we were not able to examine hippocampal subfield segmentations; this is an important future direction to investigate the specificity of these effects. In addition, because this was not an experiment, we cannot make causal or mechanistic claims about the associations between these variables. Given that we obtained the pattern of findings we predicted, future research should use more controlled experimental designs to examine these potentially causal and/or mechanistic pathways to disorder.

Although not the focus of the present study, the sample was selected on the basis of familial risk for MDD. Risk status did not moderate any of the observed associations, thus providing preliminary evidence that the associations among the variables examined in this study do not vary as a function of risk for depression. Indeed, researchers have linked environmental stress to changes in DNAm that have functional neural significance in adolescents, independent of familial risk for depression.^34^ Although we previously found MDD risk status to be associated with hippocampal volume in a partially overlapping sample,^35^ we did not find these effects in our moderation analyses. Interestingly, in that previous study we also identified significant effects of stress on volumetric reductions in the left but not right hippocampus; similar findings have been reported as sequelae of childhood maltreatment.^36^ It will be important for future research to elucidate the significance of this laterality effect as there is mixed evidence for left vs right hippocampal volume associations with chronic stress and psychopathology.^37, 38, 39, 40^

We should note a number of limitations of this study. Our findings should be considered preliminary due to the potential for false positives that are more likely in small samples and a less stringent statistical threshold used in our mediation analysis. In addition, we included only adolescent females in this study; while this increases specificity in the observed effects, our findings should be replicated in studies with more diverse samples. This is particularly important given findings of sex-specific differences in DNAm patterns in response to environmental stressors.^41^ Although this was a longitudinal study, the interval between assessments was variable and, in some cases, was quite large (up to 9 years), and we did not have measures of intervening life stress. We accounted for this variability by controlling statistically for chronological age at each measurement; nevertheless, it is important that our findings be replicated in studies with more consistent timing of measurements. While investigators have reported evidence of accelerated DNAm age in assessments conducted 9 years after the measurement of a stressor in early adolescence,^12^ DNAm age can change even within a short period of time^42^ and DNAm patterns shift rapidly during childhood and adolescence.^43^ In this context, our finding that a stress-related biomarker from early adolescence predicts hippocampal volume measured an average of 4 years later is even more striking. The change in scanner midway through the longitudinal data collection is also worth noting, although there were no systematic differences in the subcortical volumes obtained in the two scanners. We collected cortisol saliva samples, DNAm saliva samples and neural data sequentially in most of our participants; we cannot draw conclusions, however, concerning the causality of the obtained associations. Finally, although there are other epigenetic age calculators available,^5^ we were not able to compare DNAm age estimates across methods as was done in previous studies^2, 11^ because our samples were derived from saliva, and alternative methods are appropriate only for samples derived from blood.^26^ Relatedly, CpG site coverage varied from the 450 K Illumina array to the most recent EPIC chip, and 16 of the original 353 sites of the DNA Methylation Age Calculator (4.5%) were not assessed in EPIC. We should note, however, that in preliminary analyses our group has found high congruence between DNAm estimates using data from the 450 K and the EPIC platforms.

Despite these limitations, our examination of stress-related biomarkers points to mechanisms that might underlie health-related risks. Our findings indicate that cortisol levels, DNAm age and hippocampal volume are associated in a manner that implicates heightened stress affecting broad-based biological markers. This preliminary evidence of stress-related mechanisms underlying risk for mental and physical health difficulties represents an important step toward identifying targets for prevention and intervention efforts.

## References

[bib1] Zampieri M, Ciccarone F, Calabrese R, Franceschi C, Caiafa P. Reconfiguration of DNA methylation in aging. Mech Ageing Dev 2015; 151: 60–70. 25708826 10.1016/j.mad.2015.02.002

[bib2] Marioni RE, Shah S, McRae AF, Ritchie SJ, Muniz-Terrera G, Harris SE et al. The epigenetic clock is correlated with physical and cognitive fitness in the Lothian Birth Cohort 1936. Int J Epidemiol 2015; 44: 1388–1396. 25617346 10.1093/ije/dyu277 PMC4588858

[bib3] Breitling LP, Saum K-U, Perna L, Schöttker B, Holleczek B, Brenner H. Frailty is associated with the epigenetic clock but not with telomere length in a German cohort. Clin Epigenetics 2016; 8: 21. 26925173 10.1186/s13148-016-0186-5 PMC4768341

[bib4] Horvath S. DNA methylation age of human tissues and cell types. Genome Biol 2013; 14: R115. 24138928 10.1186/gb-2013-14-10-r115 PMC4015143

[bib5] Hannum G, Guinney J, Zhao L, Zhang L, Hughes G, Sadda S et al. Genome-wide methylation profiles reveal quantitative views of human aging rates. Mol Cell 2013; 49: 359–367. 23177740 10.1016/j.molcel.2012.10.016 PMC3780611

[bib6] Marioni RE, Shah S, McRae AF, Chen BH, Colicino E, Harris SE et al. DNA methylation age of blood predicts all-cause mortality in later life. Genome Biol 2015; 16: 25. 25633388 10.1186/s13059-015-0584-6 PMC4350614

[bib7] Perna L, Zhang Y, Mons U, Holleczek B, Saum K-U, Brenner H. Epigenetic age acceleration predicts cancer, cardiovascular, and all-cause mortality in a German case cohort. Clin Epigenetics 2016; 8: 64. 27274774 10.1186/s13148-016-0228-z PMC4891876

[bib8] Christiansen L, Lenart A, Tan Q, Vaupel JW, Aviv A, Mcgue M et al. DNA methylation age is associated with mortality in a longitudinal Danish twin study. Aging Cell 2016; 15: 149–154. 26594032 10.1111/acel.12421 PMC4717264

[bib9] Chen BH, Bressler J, Fornage M, Studenski S, Vandiver AR, Tanaka T et al. DNA methylation ‐ based measures of biological age: meta ‐ analysis predicting time to death. Aging (Albany NY) 2016; 8: 1–22. 27690265 10.18632/aging.101020 PMC5076441

[bib10] Gassen NC, Chrousos GP, Binder EB, Zannas AS. Life stress, glucocorticoid signaling, and the aging epigenome: Implications for aging-related diseases. Neurosci Biobehav Rev 2016; 74(Pt B): 356–365. 27343999 10.1016/j.neubiorev.2016.06.003

[bib11] Wolf EJ, Logue MW, Hayes JP, Sadeh N, Schichman SA, Stone A et al. Accelerated DNA methylation age: associations with PTSD and neural integrity. Psychoneuroendocrinology 2016; 63: 155–162. 26447678 10.1016/j.psyneuen.2015.09.020 PMC4695261

[bib12] Brody GH, Yu T, Chen E, Beach SRH, Miller GE. Family-centered prevention ameliorates the longitudinal association between risky family processes and epigenetic aging. J Child Psychol Psychiatry 2016; 57: 566–574. 26680699 10.1111/jcpp.12495 PMC4836970

[bib13] Zannas AS, Arloth J, Carrillo-Roa T, Iurato S, Röh S, Ressler KJ et al. Lifetime stress accelerates epigenetic aging in an urban, African American cohort: relevance of glucocorticoid signaling. Genome Biol 2015; 16: 266. 26673150 10.1186/s13059-015-0828-5 PMC4699359

[bib14] Carrion VG, Weems CF, Ray RD, Glaser B, Hessl D, Reiss AL. Diurnal salivary cortisol in pediatric posttraumatic stress disorder. Biol Psychiatry 2002; 51: 575–582. 11950459 10.1016/s0006-3223(01)01310-5

[bib15] Sapolsky RM. Glucocorticoids and hippocampal atrophy in neuropsychiatric disorders. Arch Gen Psychiatry 2000; 57: 925–935. 11015810 10.1001/archpsyc.57.10.925

[bib16] Lupien SJ, McEwen BS, Gunnar MR, Heim C. Effects of stress throughout the lifespan on the brain, behaviour and cognition. Nat Rev Neurosci 2009; 10: 434–445. 19401723 10.1038/nrn2639

[bib17] Vyas A, Mitra R, Shankaranarayana Rao BS, Chattarji S. Chronic stress induces contrasting patterns of dendritic remodeling in hippocampal and amygdaloid neurons. J Neurosci 2002; 22: 6810–6818. 12151561 10.1523/JNEUROSCI.22-15-06810.2002 PMC6758130

[bib18] Kaufman J, Birmaher B, Brent D, Rao U, Flynn C, Moreci P et al. Schedule for affective disorders and schizophrenia for school-age children - present and lifetime version (K-SADS-PL): initial reliability and validity data. J Am Acad Child Adolesc Psychiatry 1997; 36: 980–988. 9204677 10.1097/00004583-199707000-00021

[bib19] LeMoult J, Ordaz SJ, Kircanski K, Singh MK, Gotlib IH. Predicting first onset of depression in young girls: interaction of diurnal cortisol and negative life events. J Abnorm Psychol 2015; 124: 850–859. 26595472 10.1037/abn0000087 PMC4662047

[bib20] Pruessner JC, Kirschbaum C, Meinlschmid G, Hellhammer DH. Two formulas for computation of the area under the curve represent measures of total hormone concentration versus time-dependent change. Psychoneuroendocrinology 2003; 28: 916–931. 12892658 10.1016/s0306-4530(02)00108-7

[bib21] Horvath S, Gurven M, Levine ME, Trumble BC, Kaplan H, Allayee H et al. An epigenetic clock analysis of race/ethnicity, sex, and coronary heart disease. Genome Biol 2016; 17: 171. 27511193 10.1186/s13059-016-1030-0 PMC4980791

[bib22] Fischl B, Salat DH, Busa E, Albert M, Dieterich M, Haselgrove C et al. Whole brain segmentation: automated labeling of neuroanatomical structures in the human brain. Neuron 2002; 33: 341–355. 11832223 10.1016/s0896-6273(02)00569-x

[bib23] Fischl B, Dale AM. Measuring the thickness of the human cerebral cortex from magnetic resonance images. Proc Natl Acad Sci USA 2000; 97: 11050–11055. 10984517 10.1073/pnas.200033797 PMC27146

[bib24] Jovicich J, Czanner S, Han X, Salat D, van der Kouwe A, Quinn B et al. MRI-derived measurements of human subcortical, ventricular and intracranial brain volumes: reliability effects of scan sessions, acquisition sequences, data analyses, scanner upgrade, scanner vendors and field strengths. Neuroimage 2009; 46: 177–192. 19233293 10.1016/j.neuroimage.2009.02.010 PMC2866077

[bib25] Hayes AF. Introduction to mediation, moderation, and conditional process analysis: a regression-based approach. Guilford Publications: New York, NY, USA, 2013.

[bib26] Jones MJ, Goodman SJ, Kobor MS. DNA methylation and healthy human aging. Aging Cell 2015; 14: 924–932. 25913071 10.1111/acel.12349 PMC4693469

[bib27] Labonté B, Suderman M, Maussion G, Navaro L, Yerko V, Mahar I et al. Genome-wide epigenetic regulation by early-life trauma. Arch Gen Psychiatry 2012; 69: 113–123. 10.1001/archgenpsychiatry.2011.2287 PMC4991944 22752237

[bib28] Booij L, Szyf M, Carballedo A, Frey E-M, Morris D, Dymov S et al. DNA methylation of the serotonin transporter gene in peripheral cells and stress-related changes in hippocampal volume: a study in depressed patients and healthy controls. PLoS ONE 2015; 10: e0119061. 25781010 10.1371/journal.pone.0119061 PMC4363605

[bib29] Yang X, Ewald ER, Huo Y, Tamashiro KL, Salvatori R, Sawa A et al. Glucocorticoid-induced loss of DNA methylation in non-neuronal cells and potential involvement of *DNMT1* in epigenetic regulation of *Fkbp5*. Biochem Biophys Res Commun 2012; 420: 570–575. 22445894 10.1016/j.bbrc.2012.03.035 PMC3327767

[bib30] Chen L, Pan H, Tuan TA, Teh AL, MacIsaac JL, Mah SM et al. Brain-derived neurotrophic factor (BDNF) Val66Met polymorphism influences the association of the methylome with maternal anxiety and neonatal brain volumes. Dev Psychopathol 2015; 27: 137–150. 25640836 10.1017/S0954579414001357

[bib31] Tottenham N, Sheridan MA, Neuroscience H. A review of adversity, the amygdala and the hippocampus: a consideration of developmental timing. Front Hum Neurosci 2009; 3: 68. 20161700 10.3389/neuro.09.068.2009 PMC2813726

[bib32] Gould E, Tanapat P. Stress and hippocampal neurogenesis. Biol Psychiatry 1999; 46: 1472–1479. 10599477 10.1016/s0006-3223(99)00247-4

[bib33] Wiedenmayer CP, Bansal R, Anderson GM, Zhu H, Amat J, Whiteman R et al. Cortisol levels and hippocampus volumes in healthy preadolescent children. Biol Psychiatry 2006; 60: 856–861. 16603131 10.1016/j.biopsych.2006.02.011 PMC2367228

[bib34] Swartz JR, Hariri AR, Williamson DE. An epigenetic mechanism links socioeconomic status to changes in depression-related brain function in high-risk adolescents. Mol Psychiatry 2017; 22: 209–214. 27217150 10.1038/mp.2016.82 PMC5122474

[bib35] Chen MC, Hamilton JP, Gotlib IH. Decreased hippocampus volume in healthy girls at risk for depression. Arch Gen Psychiatry 2010; 67: 270–276. 20194827 10.1001/archgenpsychiatry.2009.202 PMC2845291

[bib36] Teicher MH, Anderson CM, Polcari A. Childhood maltreatment is associated with reduced volume in the hippocampal subfields CA3, dentate gyrus, and subiculum. Proc Natl Acad Sci USA 2012; 109: E563–E572. 22331913 10.1073/pnas.1115396109 PMC3295326

[bib37] Mervaala E, Föhr J, Könönen M, Valkonen-Korhonen M, Vainio P, Partanen K et al. Quantitative MRI of the hippocampus and amygdala in severe depression. Psychol Med 2000; 30: 117–125. 10722182 10.1017/s0033291799001567

[bib38] Bremner JD, Narayan M, Anderson ER, Staib LH, Miller HL, Charney DS. Hippocampal volume reduction in major depression. Am J Psychiatry 2000; 157: 115–118. 10618023 10.1176/ajp.157.1.115

[bib39] Papagni SA, Benetti S, Arulanantham S, McCrory E, McGuire P, Mechelli A. Effects of stressful life events on human brain structure: a longitudinal voxel-based morphometry study. Stress 2011; 14: 227–232. 21034297 10.3109/10253890.2010.522279

[bib40] Gianaros PJ, Jennings JR, Sheu LK, Greer PJ, Kuller LH, Matthews KA. Prospective reports of chronic life stress predict decreased grey matter volume in the hippocampus. Neuroimage 2007; 35: 795–803. 17275340 10.1016/j.neuroimage.2006.10.045 PMC1868546

[bib41] Essex MJ, Boyce WT, Hertzman C, Lam LL, Armstrong JM, Neumann SMA et al. Epigenetic vestiges of early developmental adversity: Childhood stress exposure and DNA methylation in adolescence. Child Dev 2013; 84: 58–75. 21883162 10.1111/j.1467-8624.2011.01641.x PMC3235257

[bib42] Boks MP, Mierlo HC, van, Rutten BPF, TRDJ Radstake, De Witte L, Geuze E et al. Longitudinal changes of telomere length and epigenetic age related to traumatic stress and post-traumatic stress disorder. Psychoneuroendocrinology 2015; 51: 506–512. 25129579 10.1016/j.psyneuen.2014.07.011

[bib43] Alisch RS, Barwick BG, Chopra P, Myrick LK, Satten GA, Conneely KN et al. Age-associated DNA methylation in pediatric populations. Genome Res 2012; 22: 623–632. 22300631 10.1101/gr.125187.111 PMC3317145

