# Supplementary material for: Accelerated DNA methylation age in adolescent girls: associations with elevated diurnal cortisol and reduced hippocampal volume
Source: Transl Psychiatry. 2017 Aug 29;7(8):e1223–. doi: 10.1038/tp.2017.188 (PMC5611751; doi:10.1038/tp.2017.188)
Supplement: Supplementary Table 1 [file tp2017188x3.docx]

Supplemental Table 1. Age of collection for each sample.

| Sample |  | Mean Age | Standard Deviation | Range |
| --- | --- | --- | --- | --- |
| Diurnal Cortisol |  | 12.52 | 1.37 | 9.12-15.03 |
| DNA Methylation |  | 12.83 | 1.50 | 10.16-16.40 |
| MRI Scan |  | 16.90 | 3.57 | 10.32-23.06 |

Note. MRI=magnetic resonance imaging.
