# Supplementary material for: Accelerated DNA methylation age in adolescent girls: associations with elevated diurnal cortisol and reduced hippocampal volume
Source: Transl Psychiatry. 2017 Aug 29;7(8):e1223–. doi: 10.1038/tp.2017.188 (PMC5611751; doi:10.1038/tp.2017.188)
Supplement: Supplementary Table 2 [file tp2017188x4.docx]

Supplemental Table 2. Participant demographics and comparison across scanners.

| Variable |  | 1.5 Tesla  (N=21) | 3 Tesla  (N=25) | Comparison |
| --- | --- | --- | --- | --- |
| Age, years (at time of scan) |  | 12.22 [2.59] | 19.16 [2.60] | *t*(44)=-6.42, *p*<.001 |
| DNAm age residual |  | -0.37 [2.61] | 0.62 [2.28] | *t*(44)=-1.37, *p*=.18 |
| Race/ethnicity (% Caucasian) |  | 67% | 56% | *χ^2^*(1)=0.55, *p*=.46 |
| SES (% household income >$100,000) |  | 53% | 47% | *χ^2^*(1)=0.11, *p*=.75 |
| High risk group (% with a mother with MDD history) |  | 38% | 64% | *χ^2^*(1)=3.07, *p*=.08 |

Note. Mean [SD]. DNAm=DNA methylation, SES=socioeconomic status, MDD=major depressive disorder.
