# Supplementary material for: Accelerated DNA methylation age in adolescent girls: associations with elevated diurnal cortisol and reduced hippocampal volume
Source: Transl Psychiatry. 2017 Aug 29;7(8):e1223–. doi: 10.1038/tp.2017.188 (PMC5611751; doi:10.1038/tp.2017.188)
Supplement: Supplementary Table 3 [file tp2017188x5.docx]

Supplemental Table 3. Comparison of key variables by familial depression risk group.

| Variable | Low Risk (N=22) | High Risk (N=24) | Comparison |
| --- | --- | --- | --- |
| Cortisol (AUCg residual) | -11.24 [139.14] | 10.92 [132.06] | *t*(44)=-0.55, *p*=.58 |
| DNAm age residual | -0.09 [2.44] | 0.40 [2.49] | *t*(44)=-0.66, *p*=.51 |
| Left hippocampal volume (residual) | -55.47 [236.58] | 50.42 [338.68] | *t*(40)=-1.16, *p*=.25 |
| Right hippocampal volume (residual) | -17.69 [282.12] | 16.22 [414.83] | *t*(40.7)=-0.33, *p*=.75 |
| Left amygdala volume (residual) | -10.24 [185.90] | 9.38 [195.86] | *t*(44)=-0.35, *p*=.73 |
| Right amygdala volume (residual) | -9.87 [231.63] | 9.05 [206.74] | *t*(44)=-0.29, *p*=.77 |

Note. Mean [SD]. AUCg=area under the curve with respect to ground, DNAm=DNA methylation.
